# Protocol: dexmedetomidine on myocardial injury after noncardiac surgery—a multicenter, double-blind, controlled trial

**DOI:** 10.1186/s13741-023-00348-6

**Published:** 2023-11-11

**Authors:** Huayue Liu, Juan Zhang, Ke Peng, Xiaowen Meng, Xisheng Shan, Wenwen Huo, Hong Liu, Yishan Lei, Fuhai Ji

**Affiliations:** 1 Department of Anesthesiology, The First Affiliated Hospital of Soochow University, Suzhou, China; 2 Institute of Anesthesiology, Soochow University, Suzhou, China; 3 Department of Pain Medicine, Shenzhen People’s Hospital (The Second Clinical Medical College, Jinan University; The First Affiliated Hospital, Southern University of Science and Technology), Shenzhen, China; 4 Department of Anesthesiology and Pain Medicine, University of California Davis Health, Sacramento, CA USA

**Keywords:** Dexmedetomidine, MINS, MACE, Myocardial injury

## Abstract

**Aims:**

Myocardial injury after noncardiac surgery (MINS) is common in elderly patients and considered as an independent predictor of 30-day mortality after noncardiac surgery. Dexmedetomidine possesses cardiac-protective profile. Previous clinical studies have found that perioperative application of dexmedetomidine is associated with decreased 1-year mortality in patients undergoing cardiac surgery. The current study protocol aims to investigate the effects of dexmedetomidine on the incidence of MINS, complications, and 30-day mortality in elderly patients subjected to noncardiac surgery.

**Methods:**

A multicenter, randomized, controlled, double-blind, prospective trial is designed to explore cardiac protection of dexmedetomidine in the elderly patients undergoing noncardiac surgery. A total of 960 patients aged over 65 years will be recruited and randomly assigned to dexmedetomidine group (group Dex) and normal saline placebo group (group NS) in a ratio of 1:1. Patients in group Dex will receive a bolus dose of 0.5 μg/kg dexmedetomidine within 10 min before surgical incision, followed by a consistent infusion at the rate of 0.3–0.5 μg/kg/h throughout the operation. Group NS patients will receive the same volume of normal saline. The primary outcome is the incidence of MINS via detecting the hs-TnT level within 3 days after the operation. The secondary outcome includes myocardial ischemic symptoms, the incidence of major adverse cardiovascular events (MACE) in hospital, length of ICU and postoperative hospital stay, the incidence of inhospital complications, and 30-day all-cause mortality.

**Discussion:**

The results of the current study will illustrate the effect of dexmedetomidine on myocardial injury for elderly patients undergoing major noncardiac surgery.

**Trial registration:**

The trial was registered with Chinese Clinical Trial Registry (CHICTR) on Aug 24, 2021 (ChiCTR2100049946, http://www.chictr.org.cn/showproj.aspx?proj=131804).

## Introduction

Annually, more than 200 million adults undergo major noncardiac surgery worldwide (Weiser et al. 2008), among which approximately 10% might experience postoperative myocardial injury characterized by a transient increase of cardiac troponin concentration in the serum (Botto et al. 2014; Devereaux et al. 2012). Although it is under controversy, researchers defined MINS (myocardial injury after noncardiac surgery) as the following: myocardial injury that may result in myocardial necrosis, is due to myocardial ischemia, has prognostic relevance, and occurs during or within 30 days after noncardiac surgery (Vascular events in noncardiac surgery patients cohort evaluation study I et al. 2019). Generally, elderly patients are more vulnerable to MINS because of poor tolerance to surgical stress and comorbid disorders. According to the VISION study, among patients aged ≥ 45 years who underwent noncardiac surgery, 17.9% was diagnosed with MINS. The incidence of MINS was 22% in another observational cohort study recruited patients ≥ 60 years old undergoing major noncardiac surgery, although most of these patients did not experience any ischemic symptoms (Waes et al. 2016).

Previous study indicated that 86–89% of perioperative high-sensitivity troponin T (hs-TnT) elevations relates to myocardial ischemia, rather than a nonischemic pathological process (i.e., sepsis or rapid atrial fibrillation) (Writing Committee for the VSI et al. 2017). Mismatch between supply and demand as well as potential coronary artery stenosis might facilitate the occurrence of MINS, especially in high-risk patients (Horr et al. 2015). Although intense monitoring is common during the perioperative stage, clinical interventions are limited in preventing the occurrence of MINS.

Dexmedetomidine, a highly selective alpha-2 (α2) adrenoreceptor agonist, provides sedation, anxiolysis, hypnosis, and analgesia effects (Chrysostomou and Schmitt 2008). It is currently widely used for sedation during perioperative period and in ICU patients. In recent years, several basic studies have shown that dexmedetomidine can significantly reduce myocardial infarction size in experimental animals with myocardial ischemia (Ibacache et al. 2012; Peng et al. 2020; Zhang et al. 2017). Our previous clinical studies found that perioperative application of dexmedetomidine was associated with a decrease in 1-year mortality and incidence of postoperative complications in patients undergoing cardiac surgery (Ji et al. 2013; Ji et al. 2014).

Based on the cardiac protective profile of dexmedetomidine and the high incidences of MINS, we presumed that dexmedetomidine might exert a beneficial effect during the perioperative period. Therefore, we design a multicenter-prospective, double-blind, randomized, and controlled trial to find out the influence of dexmedetomidine on myocardial injury of elderly patients who underwent high-risk noncardiac surgeries.

### Study design

#### Trial objectives

**Fig. 1:**
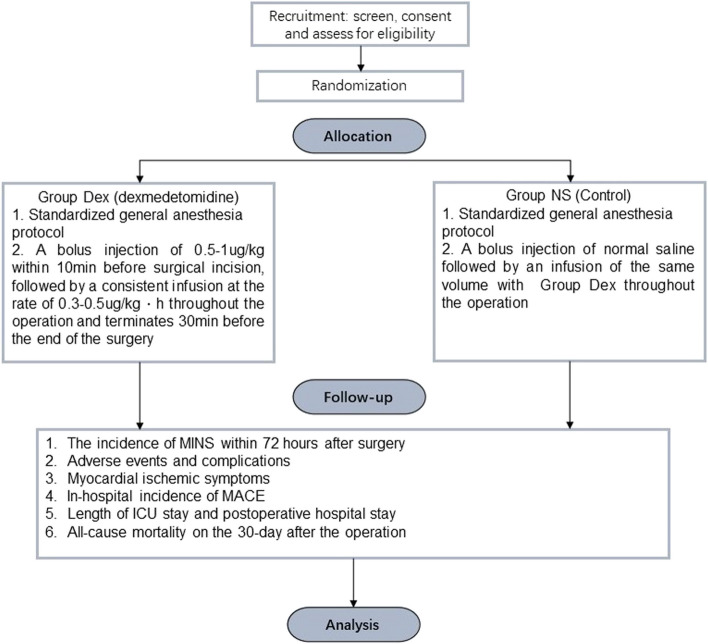
General review of study design

A multicenter, prospective, randomized, controlled, double-blind trial is designed to evaluate the influence of dexmedetomidine on elderly patients undergoing major noncardiac surgery under general anesthesia via assessing the incidence of MINS within postoperative 72 h, the adverse events, the incidence of inhospital MACE, and all-cause mortality within 30 days after the surgery. The flow of participants through the trial is summarized in Fig. 1.

### Participating centers

A total of 11 centers are going to attend this multicenter trial which is listed as the following:

1. Department of Anesthesiology, the First Affiliated Hospital of Soochow University, Suzhou, Jiangsu, China
2. Department of Anesthesiology, the Second Affiliated Hospital of Nantong University, Nantong, Jiangsu, China
3. Department of Anesthesiology, Affiliated Hospital of Nantong University, Nantong, Jiangsu, China
4. Department of Anesthesiology, Suzhou Municipal Hospital & The Affiliated Suzhou Hospital of Nanjing Medical University, Suzhou, Jiangsu, China
5. Department of Anesthesiology, the Affiliated Hospital of Xuzhou Medical University, Xuzhou, Jiangsu, China
6. Department of Anesthesiology, the First People’s Hospital of Zhangjiagang, Zhangjiagang, Jiangsu, China
7. Department of Anesthesiology, Jiangsu Cancer Hospital & Jiangsu Institute of Cancer Research and The Affiliated Cancer Hospital of Nanjing Medical University, Nanjing, Jiangsu, China
8. Department of Anesthesiology, Wuxi People’s Hospital of Nanjing Medical University, Wuxi, Jiangsu, China
9. Department of Anesthesiology, the Third Affiliated Hospital of Soochow University, Changzhou, Jiangsu, China
10. Department of Anesthesiology, Affiliated Hospital of Yangzhou University, Yangzhou, Jiangsu, China
11. Department of Anesthesiology, Zhongda Hospital affiliated to Southeast University, Nanjing, Jiangsu, China

### Inclusion and exclusion criteria

#### Inclusion criteria

1. Aged ≥ 65 years old
2. ASA I–III levels
3. Plan to perform general anesthesia with tracheal intubation for modest to high risk of elective major noncardiac surgery including gastrointestinal (including gastrectomy and enterectomy), hepatic, cystic, and pancreatic tumorectomy and thoracic (including pneumonectomy, esophageal, and mediastinum surgery), gynecologic (pelvic tumor resection), urologic (including nephrectomy and prostatectomy), and orthopedic surgeries (including arthroplasty and spine surgery).

#### Exclusion criteria

1. History of sick sinus syndrome, severe bradycardia (heart rate < 50 beats/min), left ventricular ejection fraction < 40% or heart failure, and second-degree or greater atrioventricular block without pacemaker
2. Predictive hospital stay less than 72 h
3. Severe hepatic dysfunction (Child–Pugh class C)
4. Severe renal dysfunction (requirement of renal replacement therapy before surgery)
5. New myocardial infarction within 7 days
6. Unplanned or emergent surgery
7. Pregnancy
8. Spinal and/or epidural anesthesia
9. Known allergy to α2-receptor agonist or dexmedetomidine
10. Refuse to participate in the trial

### Drop out criteria

Enrolled cases who meet any of the following criteria will be excluded for further analysis:

1. Drug is not administered.
2. No data collected
3. Suffer a second surgery

The detailed reasons for all dropout cases will be recorded, and CRFs (case report form) will be preserved for reference.

### Trial groups and procedures

#### Trial groups

Patients will be assigned into two groups according to a computer-generated randomized number table in a ratio of 1:1 of group Dex (dexmedetomidine group) and group NS (normal saline placebo group).

In group Dex (*n* = 480), patients will receive a bolus dose of 0.5 μg/kg dexmedetomidine within 10 min before surgical incision, followed by a consistent infusion at the rate of 0.3–0.5 μg/kg/h throughout the operation. The infusion was terminated 30 min before the end of the surgery.

In group NS (*n* = 480), patients will receive an infusion of the same volume of normal saline before the surgical incision and followed by a continuous intravenous infusion of normal saline with the same volume as group Dex.

#### Procedures

All intraoperative and postoperative procedures have been standardized as the following.

### Monitoring and anesthesia

The preoperative fasting time is more than 6 h. Patients do not receive preoperative medication. Standard monitoring includes the following: electrocardiogram (ECG), pulse oxygen saturation (SpO2), noninvasive blood pressure (NBP), end-tidal carbon dioxide (EtCO_2_), and bispectral index (BIS). Besides, radial artery blood pressure would be monitored, and central venus catheterization will be performed if needed.

### Anesthesia induction and maintenance

For general anesthesia induction, fentanyl 3-4ug/kg or sufentanil 0.3-0.6μg/kg, propofol 1.5-2mg/kg, and cisatracurium 0.15mg/kg will be applied followed by tracheal intubation and connection to a ventilator with 50% of inhaled oxygen concentration. Setting the tidal volume as 8-10ml/kg and the PEEP as 5-10cmH2O with the frequency of 12-15 times/min according to EtCO2 level. For anesthesia maintenance, 1-3% sevoflurane will be applied to titrate BIS value between 40-60. Additional anesthetics and sedatives will be used for maintenance if needed according to the anesthesiologists and recorded in detail. An infusion heating device will be used to maintain the nasopharyngeal temperature between 36–37°C.

The fluctuating range of blood pressure and heart rate will be maintained within ± 20%, and the intraoperative rehydration as well as the use of vasoactive drugs will be recorded. Antiemetic drugs will be injected intravenously to prevent nausea and vomiting.

### Postoperative analgesia

Multimodal analgesia apart from epidural analgesia will be used. Patient-controlled intravenous analgesia (PCIA) will be used to relieve postoperative pain.

### Evaluation and follow-up

The serum hs-TnT will be measured using the cobas e602 troponin T hs STAT assay (Roche Diagnostics, Germany). The hs-TnT level was monitored before surgery (at least 2 h before the start of surgery) and 3 days after surgery (every morning). The baseline value is the hs-TnT level measured before the operation, and the postoperative value is the highest level within 3 days after the operation. All the blood sample collected in different centers would be immediately centrifuged, and the supernatant would be extracted, frozen, and stored in a − 80 °C refrigerator. After all samples are collected, they are transported on dry ice to the First Affiliated Hospital of Soochow University for batch analysis.

The diagnostic criteria of MINS are listed as the following (Brown et al. 2017; Puelacher et al. 2018; Ruetzler et al. 2021): (1) an absolute increase of hs-TnT ≥ 14 ng/L of the postoperative value in comparison to preoperative value, (2) an increase of hs-TnT ≥ 5 ng/L above the prior concentration and with a peak of hs-TnT > 20 ng/L, and (3) any elevation ≥ 65 ng/L. If any one of the above three situations occur, it is defined as MINS.

Patients with chest tightness, chest pain, or other symptoms of myocardial ischemia, or postoperative hs-TnT value ≥ 2 times of the basic value, will be immediately performed ECG examination. Those who are diagnosed with acute coronary syndromes will immediately undergo standard medical interventions.

### Predicted adverse events in this trial and management

1. Hypotension: Systolic blood pressure < 95 mmHg or a decrease of more than 20% from baseline if the baseline value < 119 mmHg. Phenylephrine 50 μg will be given, and/or norepinephrine 0.03 μg/kg/min will be given and/or adjust the dose of anesthetics.
2. Bradycardia: Heart rate < 55 bpm or a decrease of more than 20% from baseline if the baseline value < 69 bpm. Atropine 0.25-mg and/or isoproterenol 2 μg will be given and/or adjust the anesthetics dose.

Severe adverse events (SAEs) indicate any unpredictable medical events that might prolong length of hospital stay, cause threat of life and persistent disability or dysfunction, lead to death, etc. If any SAEs occur, drug infusion will be stopped, and treatment will be initiated immediately. If there are dexmedetomidine-related death and life-threatening events, the trial will be terminated immediately, and data will be recorded in detail and carefully preserved. Besides, the events will be reported to the ethics committee as soon as possible. Severe adverse events must be followed up until it is completely resolved or when treatment is finished.

Safety outcome of this study is mortality of the patients.

### Data collection, handling, and monitoring

Preoperative and intraoperative data will be collected by the anesthesiologists who are responsible for executing research programs. The follow-up data will be recorded by researchers who are unaware of the research intervention. Details of data collection are listed in the following table (Table 1).

**Table 1:** Data collection throughout the trial

**Baseline data**
1. General information including age, gender, height, weight, BMI, ASA level
2. Hospitalization information including admission date and type and preoperative diagnosis
3. Medical history: Smoking and alcohol history, history of cardiovascular diseases and related surgery, cerebral and perivascular diseases, diabetes, and preoperative medication
4. Auxiliary examination including ECG, echocardiography, blood routine examination, biochemistry and clotting examination, serum hs-cTnT level
**Intraoperative data**
1. Surgery information: Surgery date, duration, and type
2. Anesthesia information: Total anesthetics amount, dose of vasoactive drugs, and capacity parameters (blood loss, liquid infusion, urine volume)
3. Adverse events including hypotension and bradycardia
**Postoperative data**
1. Serum hs-cTnT level and ECG with the occurrence of MINS within 72 h
2. Myocardial ischemic symptoms
1. MACE including myocardial infarction, heart block, cardiac arrest, stroke, and coma during hospital stay
2. Adverse events including hypotension and bradycardia and other complications including sepsis, pneumonia, liver dysfunction, and renal dysfunction
1. Length of ICU and postoperative hospital stay
2. All-cause mortality within 30 days

### Outcome measurements

#### Primary outcome

The primary outcome of this trial is the incidence of MINS in elderly patients undergoing major noncardiac surgery under general anesthesia within postoperative 72 h.

#### Secondary outcome

The secondary outcome includes myocardial ischemic symptoms, the incidence of MACE (myocardial infarction, heart block, cardiac arrest, stroke, coma) inhospital, length of ICU stay, length of postoperative hospital stay, the incidence of sepsis, pneumonia, liver dysfunction, renal dysfunction inhospital, and 30-day all-cause mortality.

### Randomization, blinding, allocation, and concealment

This trial is multicenter double-blinded, randomized, prospective, parallel controlled design. The participants, anesthesiologists, surgeons, and investigators are responsible for data collection, and other healthcare providers will remain masked to treatment allocation until the completion of final analysis.

1. Randomization is implemented by using a computer-generated randomized number serials (SAS 9.2) with a block size of 4. The results of randomization will be sealed in consequently numbered envelops and stored at the site of investigation.
2. The recruited patients are blind to the randomized allocation.
3. The research coordinator is responsible for opening the randomization envelope and coordinating research.
4. Study drugs (dexmedetomidine chloride 200 μg/2 mL and normal saline 2 mL) will be provided as clear aqueous solutions in the same 3-mL bottles. A pharmacist who does not participate in the rest of the study extracts 2 mL of the study drug and dilutes it to 50 mL (dexmedetomidine chloride 4 μg/mL). All injection pumps and syringes used for study drug administration were same in external appearance. The pharmacist will encode the study drugs according to the randomization sequence.
5. Anesthesiologists collect the preoperative and intraoperative data of the participants and use the distributed experimental drugs to execute research programs.
6. The other two researchers who are blind to the randomized allocation of patients will be responsible for collecting the postoperative follow-up results data. The anesthesiologist and the researchers do not know the each other’s collected results.
7. The statistical analysis will be carried out independently by the appointed statistician who is blind to the randomized allocation of patients.
8. When all cases complete, all data are inputted into the electronic data capture (EDC) and checked without mistakes. The blindness will be unmasked, and the database will be locked up. The database will be sent to the independent biostatistician for statistical analysis.

### Study sample calculation

According to the previous study, the incidence of myocardial injury is about 22.2% in elderly patients after major noncardiac surgery (Waes et al. 2016). We assume that the incidence of MINS will be reduced by 1/3 (to 14.8%) in the dexmedetomidine group. We calculate the required sample size with *Z*-test (PASS 15.0 software) with a significance and power of 0.05 (two-sided) and 80%, respectively, and the sample size required to detect differences is 864 patients in all. Taking into account a loss-to-follow-up rate of about 10%, we plan to enroll 960 (480 in each group) patients in this study.

### Data management and statistical analysis

#### Data management

1. Investigators should correctly, promptly, and completely record data in the standard data collection and management system including a CRF and an electronic data capture (EDC) according to original observation.
2. Data input will be performed by one researcher and checked out by another independent investigator.
3. Data management will be inspected by the Clinical Research Ethics Committee of the First Affiliated Hospital of Soochow University.

### Statistical analysis

#### General principles

Categorical variables will be presented as number of cases (percentage). Numeric variables will be presented as mean (standard deviation) or median (maximum, minimum, or interquartile range). The two-sided test will be used in all statistical analysis, and *p*-values of less than 0.05 will be statistically significant (unless otherwise indicated).

#### Patient recruitment and drop-out status

The status of participants recruitment and dropout will be listed and summarized. Comparison of the all drop-out rate between the two groups will be performed with chi-square test.

#### Demographics and baseline characteristics

Demographic data and baseline characteristics (such as previous history of medication and comorbidity) will be recorded. Comparison of baseline numeric variables (such as BMI) between groups will be performed with Wilcoxon rank-sum test or independent sample *T*-test. Comparison of categorical variables (such as presence of a comorbidity, gender) between groups will be performed with Fisher exact test or chi-square test.

#### Effectiveness evaluation

##### Evaluation of primary endpoint

The incidence of MINS within 3 days after surgery will be calculated. Comparison between groups will be performed with chi-square test. The logistic regression analysis will be performed to evaluate risk for postoperative MINS with odds ratio (OR) and 95% CI as measures of association.

##### Evaluation of secondary endpoints

Survival analysis and difference between groups assessed by log-rank test will be used to analyze time-to-event variables (length of stay in ICU, length of stay in hospital after surgery). Chi-square test will be used to compare the incidence of MACE and postoperative complications in hospital and 30-day mortality.

##### Sensitivity analysis

Perioperative variables and baseline that differed between groups (*p* < 0.05) together with the intervention drug (dexmedetomidine or NS) will be entered into a multivariate logistic regression analysis model against the primary outcome (postoperative MINS or not), in order to adjust for their potential confounding effects on the primary outcome.

#### Interim analysis

**Fig. 2:**
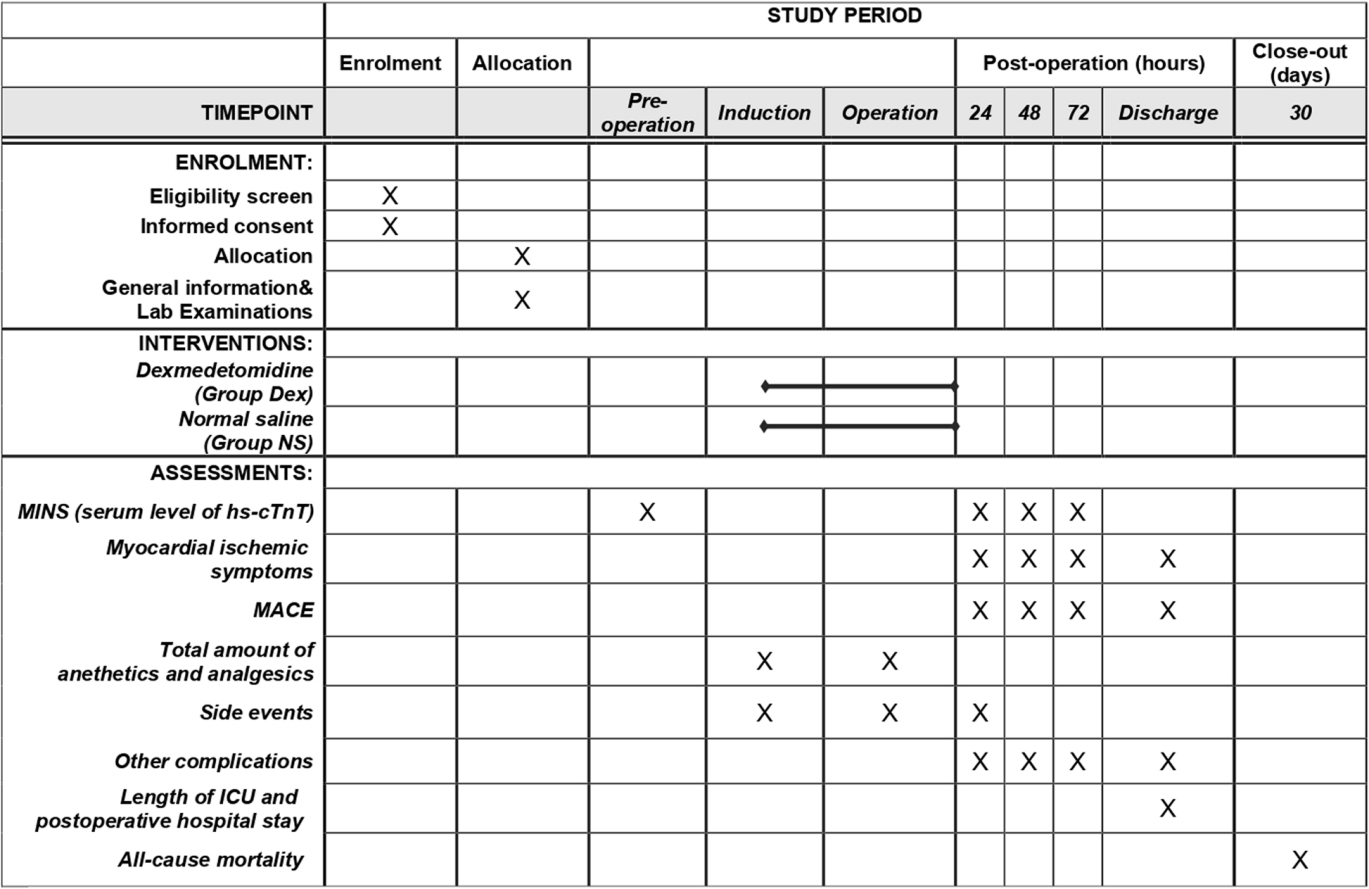
Study timeline and schedule of enrolment, allocation, interventions, and assessments according to SPIRIT 2013 statement

An independent statistician will implement a planned interim analysis after half of the total patients recruited. Interim analysis will provide a possibility for sample size recalculation and early stop for the trial. An overview of the study protocol complies with the Standard Protocol Items: Recommendations for Interventional Trials (SPIRIT) guidelines and is presented in Fig. 2.

### Study termination

1. If a study drug-related death occurs during the trial period, the study must be stopped. A report must be sent to the ethics committee. Restart of the trial should need a new approval from the ethics committee.
2. The trial will be terminated after accomplishment of patient recruitment and all data collection. Decision will be made by the principal investigator.

### Trial registration

The trial was registered with Chinese Clinical Trial Registry (CHICTR) on Aug 24, 2021 (ChiCTR2100049946, http://www.chictr.org.cn/showproj.aspx?proj=131804). We started recruiting participants after receiving the registration number.

## Discussion

In this multicenter, randomized double-blind, prospective, parallel controlled trial, we intend to evaluate the effects of dexmedetomidine on major adverse cardiovascular events and 30-day outcomes in elderly patients ≥ 65 years old who will undergo modest to high risk of noncardiac surgery. Our primary hypothesis is that perioperative dexmedetomidine application would reduce the incidence of MINS in elderly patients undergoing major noncardiac surgery under general anesthesia within postoperative 72 h. The second hypothesis is that dexmedetomidine would alleviate the incidence of MACE (i.e., myocardial infarction, heart block, cardiac arrest, stroke, coma) inhospital, reduce 30-day all-cause mortality, and benefit other outcomes. The reporting and implementation of this trial will follow the Consolidated Standards of Reporting Trials (CONSORT) guidelines (Moher et al. 2010).

Myocardial injury is common in patients older than 65 years old after noncardiac surgery. According to previous studies, MINS has been demonstrated as an independent predictor of 30-day mortality after noncardiac surgery (Botto et al. 2014; Waes et al. 2013). Besides, MINS indicates an increased risk of 1-year-all-cause mortality. Considering the potential risks for patients who undergo noncardiac surgeries, especially for elderly patients with multiple combinations, the 2016 Canadian Cardiovascular Society (CCS) guidelines recommended daily postoperative troponin measurements for 48–72 h in patients with high cardiac risk index scores (class 1 recommendation, level B evidence) (Duceppe et al. 2017). Myocardial injury after noncardiac surgery is strongly associated with mortality, even in the absence of electrocardiographic changes, clinical symptoms, or imaging evidence of myocardial ischemia consistent with myocardial infarction (Devereaux and Szczeklik 2020).

Dexmedetomidine, a highly selective α­2 adrenergic agonist, is commonly used for sedation. Dexmedetomidine minimally impairs ventilation (Keating 2015). For patients aged over 65 years old, prophylactic low-dose dexmedetomidine significantly decreases the incidence of delirium within 7 days postoperatively (Su et al. 2016). Our previous study found that perioperative dexmedetomidine infusion was associated with improved 5-year survival in patients undergoing cardiac surgery (Peng et al. 2021). Moreover, the data from our animal experiments (Zhang et al. 2017; Zhang et al. 2019) indicated dexmedetomidine can alleviate myocardial injury and inflammation induced by myocardial ischemia and reperfusion, in which the cholinergic anti-inflammatory pathway may be involved. Although a meta-analysis of randomized trials from Jin et al. (Jin and Zhou 2017) found no significant differences between dexmedetomidine and placebo on the outcomes including all-cause mortality, myocardial infarction, and myocardial ischemia incidences in noncardiac surgery, it still lacks of a large-sampled randomized trial especially in high-risk patients. In this study, we will use the dexmedetomidine perioperatively aiming to reduce the incidence of MINS and improve the outcomes in noncardiac surgery.

This study has several strengths. To the best of our knowledge, this will be the first multicenter, randomized, double-blind, prospective, comparative effectiveness trial with an adequate power to assess the impact of perioperative dexmedetomidine management strategies on the occurrence of myocardial injury and major complications in elderly patients undergoing major noncardiac surgery. Xu et al. (Xu et al. 2014) found that dexmedetomidine can reduce myocardial injury in patients undergoing noncardiac surgery, but it is a single-center, small sample trial. In addition, we use preoperative and postoperative hs-cTnT measurements to detect MINS. In a separate single-center prospective study (Puelacher et al. 2018), MINS was defined as an absolute change of hsTnT > 14 ng/L and associated with 30-day mortality. Besides, this study will collect postoperative complications as well as 30-day mortality.

This study also has some limitations. This study measured hs-cTnT routinely in the first 3 days after surgery and afterward only in case of clinical suspicion. In addition, we include patients aged over 65 years old with ASA I–III levels, and thus, the influence of dexmedetomidine strategies on patients who are sicker (i.e., ASA status IV) needs further studies.

In conclusion, the current study aims to illustrate the effect of dexmedetomidine on myocardial injury for elderly patients undergoing major noncardiac surgery. We will report the results according to the CONSORT checklist. We expect that perioperative dexmedetomidine application would decrease incidence of MINS and mortality in patients undergoing noncardiac surgery.

## Data Availability

Data sharing is not applicable to this article as no datasets were generated or analyzed during the current study.

## Abbreviations

MINS: Myocardial injury after noncardiac surgery
hs-TnT: High-sensitivity troponin T
MACE: Major adverse cardiovascular events
ECG: Electrocardiogram
SpO2: Pulse oxygen saturation
NBP: Noninvasive blood pressure
EtCO_2_: End-tidal carbon dioxide
BIS: Bispectral index
SAEs: Severe adverse events
EDC: Electronic data capture
